# Toxic Habits: An Analysis of General Trends and Biases in Snake Venom Research

**DOI:** 10.3390/toxins14120884

**Published:** 2022-12-17

**Authors:** Ignazio Avella, Wolfgang Wüster, Luca Luiselli, Fernando Martínez-Freiría

**Affiliations:** 1 CIBIO, Centro de Investigação em Biodiversidade e Recursos Genéticos, InBIO Laboratório Associado, Campus de Vairão, Universidade do Porto, 4485-661 Vairão, Portugal; 2 Departamento de Biologia, Faculdade de Ciências, Universidade do Porto, 4099-002 Porto, Portugal; 3 BIOPOLIS Program in Genomics, Biodiversity and Land Planning, CIBIO, Campus de Vairão, 4485-661 Vairão, Portugal; 4 Molecular Ecology and Evolution at Bangor, School of Natural Sciences, Bangor University, Bangor LL57 2UW, UK; 5 Institute for Development, Ecology, Conservation & Cooperation, Via G. Tomasi di Lampedusa 33, I-00144 Rome, Italy; 6 Department of Zoology, University of Lomé, Lomé 01BP1515, Togo; 7 Department of Environmental and Applied Biology, Rivers State University of Science and Technology, Port Harcourt P.M.B. 5080, Nigeria

**Keywords:** snakes, venom, trends, bibliometric analysis, research biases, review

## Abstract

Biases in snake venom research have been partially identified but seldomly quantified. Using the Google Scholar web search engine, we collected a total of 267 articles published between 1964 and 2021, and reviewed them to assess the main trends in this field of study. We developed a 4-category classification of the harmful potential of each of the 298 snake species retrieved from the analysed publications, and tested whether taxonomy, realm of origin, and/or assigned hazard category could affect how often each of them appeared in the articles considered. Overall, viperids were significantly more represented than any other snake taxon retrieved. The Neotropics were the most represented biogeographic realm for number of studied species, whereas information about the country of origin of the analysed specimens was often incomplete. The vast majority of the publications focused on snake venom characterisation, whereas more ecology-related topics were rarely considered. Hazard category and biogeographic realm of origin of each species had a significant effect on the number of articles dedicated to it, suggesting that a snake’s harmful potential and place of origin influence its popularity in venom studies. Our analysis showed an overall positive trend in the number of snake venom studies published yearly, but also underlined severe neglect of snake families of supposedly minor medical relevance (e.g., Atractaspididae), underrepresentation of some of the areas most impacted by snakebite (i.e., Indomalayan and Afrotropic realms), and limited interest in the ecological and functional context of snake venom.

## 1. Introduction

About 200,000 species of venomous animals belonging to many different taxa (e.g., cnidarians, arthropods, reptiles, fishes, mammals) are currently known to science [1]. Among them, snakes are arguably the most notorious ones. Of more than 3900 snake species currently recognised [2], about 300, mainly belonging to the families Viperidae (e.g., adders, rattlesnakes, palm pit vipers), Elapidae (e.g., cobras, coral snakes, sea snakes), and Colubridae (e.g., vine snakes, twig snakes, boomslangs) are considered of medical importance by the World Health Organization (WHO) [2,3,4].

Snake venom is a complex mixture of peptides, proteins, small organic molecules, and salts [5,6], able to disrupt the general homeostasis of the envenomated organism, affecting it in different ways and with different levels of specificity and potency [7,8,9]. Snake venom has long been and remains in the spotlight for researchers from all over the world, mostly because of the medical importance of snakebites for human health [10,11,12]. For many years, it has been studied through traditional biochemical and pharmacological approaches, often focusing mainly on abundant toxins present in venoms produced by the most common medically relevant species (e.g., *Daboia russelii* [13], *Bothrops jararaca* [14]), and neglecting the venoms of a large number of rarer and/or generally less studied species [15,16,17].

Advances in the so-called “omic” technologies, defined as the application of high-throughput methodologies [18], and their inclusion in the conventional analysis procedures, completely revolutionised snake venom studies. The term “venomics” currently describes the comprehensive study and characterisation of the whole venom profile of a toxic organism by the means of integrated “omic” methodologies, namely proteomics, transcriptomics, and genomics [5,18,19]. Specifically, modern snake venomics allow for the identification of venom components both directly, through the analysis of the protein content of crude venom (i.e., proteomics), and indirectly, through the sequencing of venom gland mRNA or cDNA (i.e., transcriptomics), or of the full genome (i.e., genomics) of the analysed species [19]. By allowing the rapid characterisation of all venom components of a growing number of snake species from all over the world, the deployment of snake venomics is gradually filling the knowledge gap left by the application of previous, less comprehensive approaches. The remarkable amount of information produced by venomics about composition and properties of different snake venom phenotypes, together with its implementation in functional studies, is helping to elucidate the processes underlying snake venom origin and evolution, and is driving the development of powerful therapeutic tools to be used to mitigate the global burden of snakebite [20,21] and successfully treat different types of diseases (e.g., [22,23] and references within).

Despite the growing attention snake venom studies have received since the rise of venomic approaches, it has been noted that, in this line of research, some topics tend to be investigated more frequently than others [5,24,25]. Questions regarding snake ecology (e.g., interactions between snakes and their prey), for example, are seldom addressed in venomic studies, despite being relevant for both evolutionary biology and the development of effective snakebite mitigation strategies [15,26,27]. Similarly, research efforts seem to be greatly biased towards species belonging to the families Elapidae and Viperidae, whereas other snake families (e.g., Psammophiidae, Pseudoxyrophiidae, Atractaspididae, Homalopsidae) tend to be overlooked [15]. Although these tendencies in snake venom studies have long been recognised (e.g., [15,28]), they have never been formally quantified.

In the present work, we aim to provide formal categorisation and quantification of the current biases in snake venom research. To this end, we (i) present an estimation and description of the prevailing trends in snake venom studies published between 1964 and 2021, (ii) analyse whether and how the focus of the retrieved studies changed in terms of topics and taxa covered across the defined time frame, and (iii) test whether potential biases in terms of number of articles dedicated to each retrieved snake species could be related to specific factors (e.g., taxonomy, biogeographic realm of origin). We expect our findings to uncover the taxonomic and topic imbalances present in this field of study, and potentially help identify their origin and define the directions to follow to redress them.

## 2. Results

### 2.1. Taxonomic Information

A total of 267 articles was considered for the current study (complete list provided in Table S1). From the analysis of these articles, we gathered information about a total of 298 snake species belonging to seven different families: Viperidae, Elapidae, Colubridae, Atractaspididae, Homalopsidae, Psammophiidae, and Pseudoxyrhophiidae. Most of the retrieved species belonged to the families Viperidae and Elapidae.

**Figure 1:**
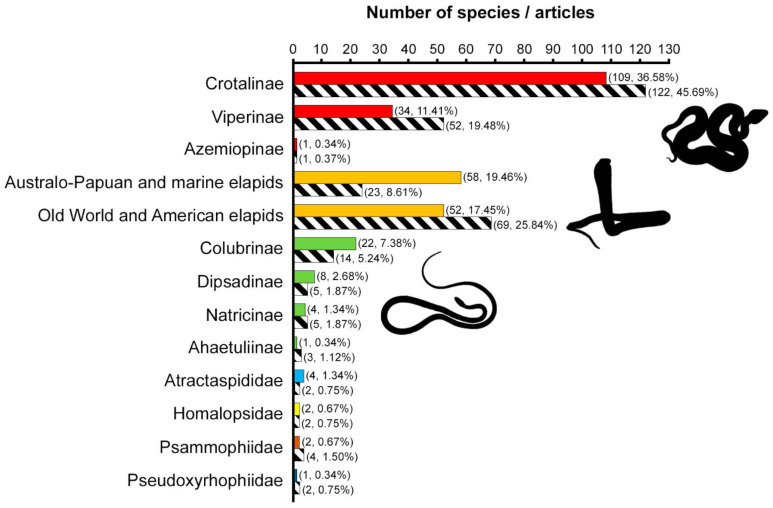
Number of species and articles for the snake families and subfamilies studied in the reviewed publications. Coloured bars refer to the number of species belonging to each taxon as retrieved from the analysed publications; striped bars refer to the number of gathered articles studying members of each taxon. Exact numbers are reported in parentheses. The percentages refer to the total number of species and publications retrieved. Bars of the same colour correspond to subfamilies belonging to the same family (i.e., red = Viperidae, orange = Elapidae, green = Colubridae). Following [2], for the families Atractaspididae, Homalopsidae, Psammophiidae, and Pseudoxyrhophiidae, no subfamilies are currently identified. Original elapid silhouette by Chris Hay, provided via www.phylopic.org, and modified and used under license CC BY-NC 3.0.

The differences in article coverage of snake families and subfamilies/groups were significant (families: *χ*^2^_(6)_ = 243.1, *p* < 0.0001; subfamilies/groups: *χ*^2^_(12)_ = 228.9, *p* < 0.0001). The family Viperidae was the most studied one, followed by the families Elapidae and Colubridae. Most of the viperids belonged to the subfamily Crotalinae, which was the most studied snake subfamily. In terms of number of articles and studied species, the snake families Atractaspididae, Homalopsidae, Psammophiidae, and Pseudoxyrhophiidae were both the least studied and the least represented. For further details about the number of species and articles retrieved for each family and subfamily/group, see Figure 1.

**Figure 2:**
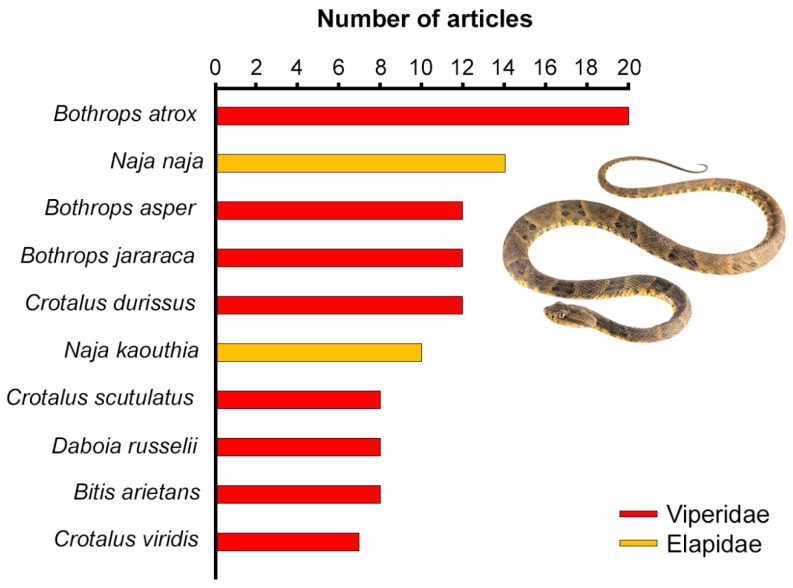
Information about the top ten most studied snake species. The graph shows the number of retrieved articles studying each species. Depicted in photo, *Bothrops atrox* (edited from [29]).

A total of 96 snake genera were retrieved from the literature analysis, mostly belonging to the families Elapidae (35 genera, 36.46%), Viperidae (30 genera, 31.25%), and Colubridae (25 genera, 26.04%). The differences in article coverage between snake genera were significant (*χ*^2^_(95)_ = 196.7, *p* < 0.0001). With 26 species studied in the considered publications, *Bothrops* was the most represented genus, followed by *Crotalus* (24 studied species) and *Micrurus* (19 studied species). *Bothrops* and *Crotalus* were also the two most studied genera (43 and 39 articles, respectively). The most studied species overall was the South American pit viper *Bothrops atrox* (20 publications, 7.49% of all retrieved articles; Figure 2). The complete list of all retrieved snake taxa is reported in Table S2.

### 2.2. Hazard Categories

Most of the 298 snake species retrieved from the analysed literature were assigned to the first two hazard categories. Specifically, most of viperids and elapids were considered of critical clinical relevance/category 1 or high clinical relevance/category 2. The species considered of moderate clinical relevance/category 3 and low clinical relevance/category 4 encompassed most of the Colubridae, and all of the Homalopsidae, Psammophiidae, and Pseudoxyrhophiidae species retrieved). Because of the lack of data about envenomation caused by the elapid *Toxicocalamus longissimus*, this species was the only one we were unable to assign to any on the four hazard categories defined. For detailed information about the number of species included in each hazard category, and the hazard category assigned to each one of them, see Table 1 and Table S2.

### 2.3. Countries and Biogeographic Realms

**Figure 3:**
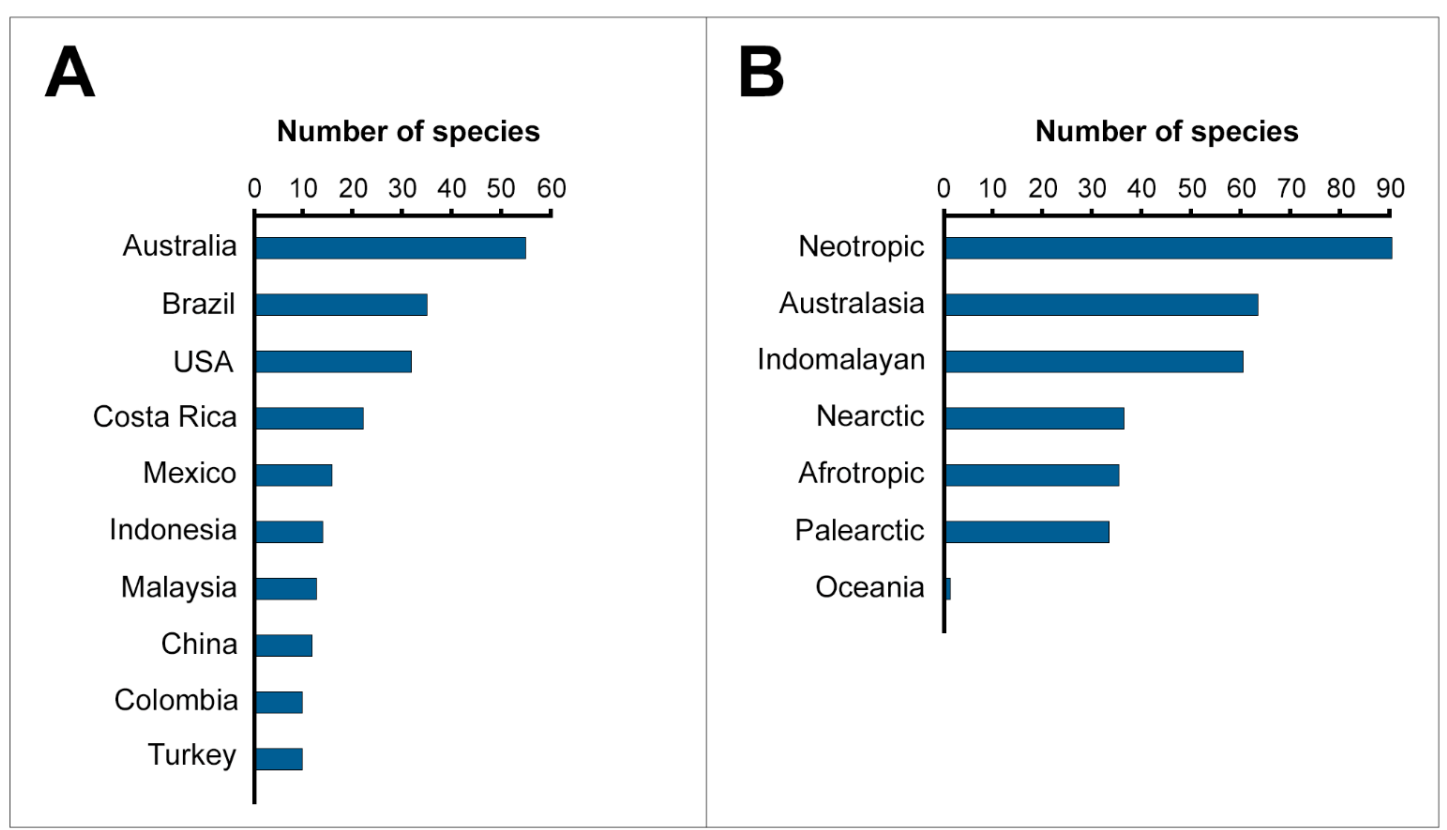
Information about the geographic origin of the species retrieved from the analysed publications. The graphs show the number of species recorded for the ten most represented countries (panel **A**) and biogeographic realms of origin (panel **B**).

The most represented country in terms of number of studied species was Australia (55 species), followed by Brazil (35 species), and the USA (31 species; Figure 3A). The country of origin of 42 specimens belonging to 34 different species was impossible to identify based on the information reported in the publications. Detailed information about the number of species retrieved for each country is reported in Table S3. Overall, the frequency of papers by country was significantly biased (*χ*^2^_(75)_ = 217.6, *p* < 0.0001), and Australia exceeded all other countries in terms of number of published studies. Since information about the country of origin of the retrieved species was generally more incomplete than the information about the biogeographic realms, we excluded it from further analysis.

The most represented biogeographic realm was the Neotropics (89 species), followed by the Australasia and the Indomalayan realms (60 species each; Figure 3B). The biogeographic realm of origin of six specimens belonging to five different species could not be identified (see Table S1). The differences in number of species studied for the various realms were statistically significant (*χ*^2^_(6)_ = 66.1, *p* < 0.0001).

### 2.4. Topics Investigated

**Figure 4:**
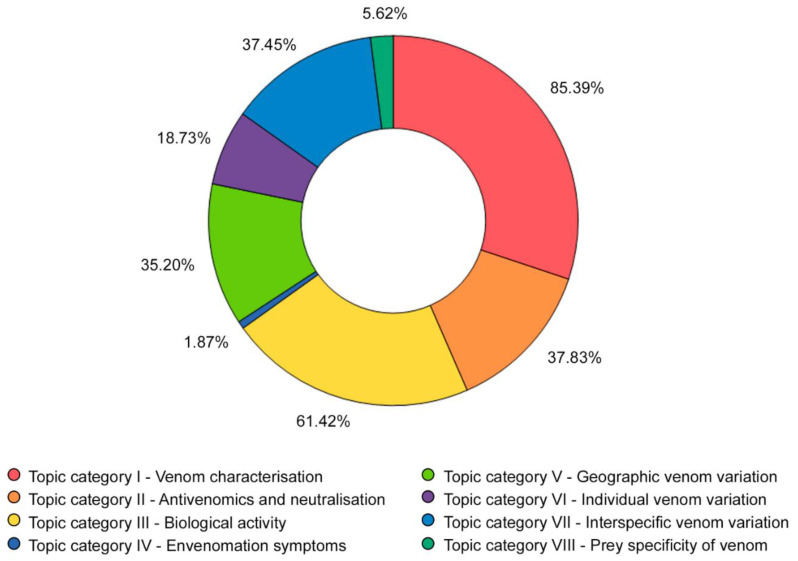
Information about the topics investigated in the reviewed articles. The graphs show the percentages of articles covering each of the eight topic categories defined. The results about topic cover are based on added up values and each topic has been counted separately; therefore, the overall sum of resulting percentages is greater than 100%.

None of the articles covered all the eight defined topic categories. The highest number of topics covered by one article was five (eight articles, 3% of the total), whereas the lowest was one (23 articles, 8.61% of the total). Most of the articles (110 articles, 41.2% of the total) covered three topic categories. The most covered topic was “venom characterisation” (228 articles), whereas the least covered one was “envenomation symptoms”, studied in only five publications. The most studied combination of topics was “venom characterisation + interspecific venom variation” (Figure S1), found in 24 articles. The differences in number of articles covering each topic category were significant (*χ*^2^_(7)_ = 225.9, *p* < 0.0001). Further information about the topics investigated in the analysed publications is reported in Figure 4.

### 2.5. Chronological Trends

Our analysis performed on articles published between 1964 and 2021 showed that the number of publications on snake venom increased significantly and non-linearly over the years, with the best fitting model curve [y = yearly number of studies; x = years passed since the beginning of the survey (i.e., 1964)] being a 3rd-order polynomial fit (yearly number of articles = 0.003654x^3^ − 21.72x^2^ + 4.303 × 10^4^x − 2.842 × 10^7^; AICc = 2199, *χ*^2^ = 2190, r^2^ = 0.9942, *p* < 0.000001). A conspicuous increase in number of articles published each year was detected starting in the early 2000s, with most of the retrieved articles (240 articles, 89.9% of the total) being published after 1995. Considering the whole timeframe, between 4 and 5 articles were published on average every year. The number of gathered articles reached double digits for the first time in 2006 (11 articles, 4.12% of the total) and its peak in 2019 (29 articles, 10.9% of the total).

#### 2.5.1. Taxonomic Information: Snake Families and Subfamilies

**Figure 5:**
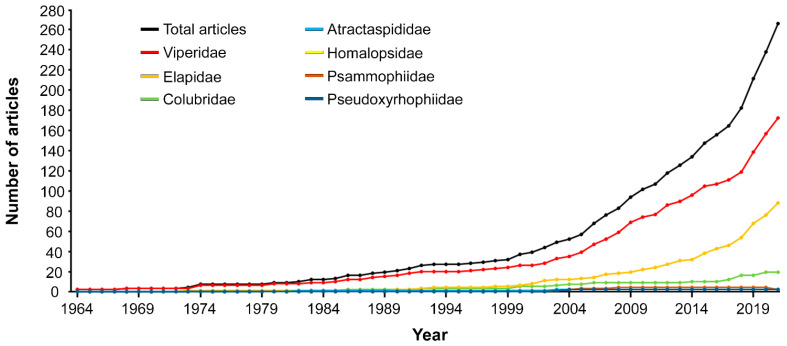
Chronological distribution of the analysed articles by snake families. The number of articles analysed per each year of the defined time frame is reported in Table S4. The black cumulative curve shows the overall positive trend of the analysed articles along the considered timeframe.

Notably, the number of articles dedicated to members of the families Viperidae and, to a lesser degree, Elapidae, has been growing at a particularly fast pace, outdistancing the curves relative to other families already in the late 90s (see Figure 5). The beginning of the twenty-first century also marked an increase in the curve relative to the family Colubridae, which however remained relatively low. Along the considered timeframe, the families Atractaspididae, Homalopsidae, Psammophiidae, and Pseudoxyrhophiidae were confirmed to be consistently less studied than the families Viperidae, Elapidae, and Colubridae, and did not experience any significant increase in number of articles (see Figure 5).

Overall, the number of articles focusing on elapid and viperid subfamilies increased since the second half of the first decade of the 2000s (see Figure S2). The number of articles focusing on Crotalinae was lower than the number of articles focusing on the other viperid subfamily Viperinae until the mid-1980s, but then increased to the point of largely outdistancing all the others. The curve corresponding to Old World and American elapids showed a very similar pattern (see Figure S2). Among colubrids, Colubrinae was the most studied subfamily, and underwent a slight increase in articles in the last three years (Figure S2). The subfamilies Azemiopinae, Ahaetuliinae, Dipsadinae, and Natricinae were consistenly the least studied ones across the whole timeframe.

#### 2.5.2. Topics Investigated

**Figure 6:**
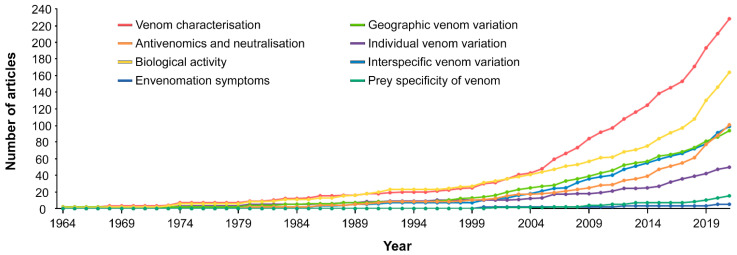
Chronological distribution of topic cover in the analysed articles. The cumulative curves show the trend of the analysed articles along the considered timeframe by topic category.

A general increase in publications can be detected from the beginning of the 2000s (see Figure 6). The chronological trend of articles focusing on “biological activity” showed a moderate growth until the early 2000s, and then underwent a considerable rise. A similar trend was detected for the studies dealing with the topic “antivenomics and neutralisation”. The curves relative to the topics “geographic venom variation” and “interspecific venom variation” were almost overlapping throughout the considered time frame (Figure 6). A significant increase was evident in the number of yearly papers concerning the former topic category, and an almost exponential by-year increase in the number of papers dedicated to the latter was observed. We also detected a significant growth throughout the years in the number of published studies focusing on the topic “venom characterisation”, obtained exactly for the overall number of publications. The chronological trend concerning articles focusing on the topic “individual venom variation”, although significant, was less evident.

Although a certain degree of convergence between the positive trajectories followed by the curves relative to the abovementioned topic categories was evident, other subjects did not show such defined growths along the whole time frame considered. Specifically, because of the extremely small number of retrieved publications concerning “envenomation symptoms” and “prey specificity of venom”, we were unable to detect any marked chronological trend in terms of yearly number of articles focusing on these two topic categories.

Details of the statistical analyses performed are reported in Table S5.

### 2.6. Factors Influencing the Differences in Number of Articles between Species

The model that best described the variation in number of articles dedicated to each snake species included the biogeographic realm and hazard category as independent variables (see Table 2). The effect of the hazard category assigned to each species was statistically significant (*χ*^2^_(3)_ = 59.8; *p* < 0.001), as well as the effect of the biogeographic realm of origin of each species (*χ*^2^_(5)_ = 21.5; *p* < 0.001).

## 3. Discussion

### 3.1. Viperids Are the Most Studied Snakes

From the analysis of all the considered publications, members of the family Viperidae were the most studied both in terms of number of species investigated and articles (Figure 1). Among the factors we tested to try to understand what could determine the prevalence of viperids, and more in general the disproportion in terms of number of articles between the studied snake taxa, the hazard categories we assigned to the species were statistically significant. Specifically, the results of our analysis showed that the number of articles focusing on species of critical clinical relevance/category 1 is significantly higher than the number of articles focusing on species with a lower hazard rating (see Table S6). Assuming that the hazard index we defined is able to accurately represent the danger a snake species can pose to humans because of its venom, our findings suggest that one of the main criteria used to select which snake venom to analyse might be its noxious potential. This is supported by the fact that snakes belonging to the three most studied snake families (i.e., Viperidae, Elapidae, Colubridae) are the ones most frequently and notoriously involved in snakebite accidents [4,30,31].

Viperids, widely distributed and highly diverse [2], have indeed a major impact on human health in terms of snakebite, being responsible for a large number of bites and deaths in the three global snakebite hotspots (i.e., Africa, Asia, and Central and South America [32,33,34]. Within Viperidae, the subfamily Crotalinae (i.e., pit vipers, exclusive to America and Asia) was the most studied subfamily overall. In the American continent, pit vipers cause the great majority of snakebite accidents, which are usually characterised by higher morbidity and mortality than those caused by their Asian counterparts [35,36]). In particular, species belonging to the genus *Bothrops*, the most studied genus across the analysed articles, account for 50–80% of all the snakebite accidents happening in most countries of Latin America [30]. In this scenario, it appears plausible that the prevalence of pit vipers throughout the considered publications might be related to their perceived harmfulness and relevance for human health.

Elapid snakes are generally accountable for fewer ophidic accidents than viperids in the American continent (e.g., [30]), but are of extreme medical importance in Asia and Africa (e.g., [4,33,34]). We hypothesise that the detected disproportion in terms of article coverage between elapids and viperids might be related to factors different from those we accounted for in our analysis. Specifically, we speculate that this disproportion to be due to socio-economic factors, namely research on snake venom being generally less developed in most Asian and African countries [37,38], resulting a lower number of studies focusing on species originating from these areas. Conversely, we suspect the underrepresentation of Atractaspididae, Colubridae, Homalopsidae, Psammophiidae, and Pseudoxyrhophiidae in the analysed articles to be mainly caused by these families being typically considered of only minor medical relevance, and to the general lack of detailed information about the danger many of their members can pose to humans [39,40].

### 3.2. The Neotropics as a Gold Mine for Snake Venom Studies

In line with the bias towards American pit vipers mentioned earlier, the most represented biogeographic realm was the Neotropics (Figure 3B). Encompassing Central and South America, it is home to about 900 snake species [2,41]. As a consequence of this remarkable ophidian diversity, the Neotropics are a global hotspot of medically important snake species, for many of which no effective therapy is listed by the WHO (like the Congo Basin and southeast Asia [3]), and which pose a serious threat to the large part of the local population, leading a markedly rural lifestyle [12,42,43]. In recent years, various research centres and laboratories located in Central and South America (e.g., Instituto Butantan in Brazil, Instituto Clodomiro Picado in Costa Rica) have made a remarkable contribution to snakebite studies [37,38], largely focusing on local medically relevant snake species. In light of this, we suspect that the prevalence of the Neotropics in terms of number of studied snake species might be determined not only by this realm’s abundance of species relevant for snakebite and snake venom research, but also by the large number of studies developed by Central and South American institutions included in our analysis (see Table S1).

In spite of the evident prevalence of Neotropical snakes in the analysed publications, the effect of the Neotropics on the number of articles dedicated to each retrieved species was generally non-significant. In fact, compared to species originating from the Neotropics, only the Australasian species appear to be significantly underrepresented in terms of number of articles (*B* ± *SE* = −0.386 ± 0.133; *p* = 0.004; Table S6). From this perspective, it is interesting to note that in our analysis, species originating from Palearctic, Nearctic, and Indomalayan realms appear to perform positively in terms of number of articles when compared to Neotropical species, although non-significantly (see Table S6). Although we found the biogeographic realm of a snake species to have an overall significant effect on the number of articles focusing on it, these results suggest that the success of Neotropical species in snake venom research is not strictly because of their realm of origin, thus supporting the role of the danger a snake species can pose to humans as one of the main factors determining a species’ popularity in snake venom studies.

### 3.3. The Neglect of the Ecological Context

The characterisation of the compounds present in snake venom is a crucial step for a wide spectrum of studies, from those focusing mainly on its biological and evolutionary significance to those focusing on snakebite management and antivenom testing [44,45,46]. The same considerations can be made for the analysis of the biological activity of snake venom, relevant in venom variation investigation [47] and comparative research (e.g., [48,49]), and fundamental for the study of the pathophysiological effects of envenomations. The publications we collected and analysed encompass a considerable part of this spectrum, which could thus possibly explain the significant prevalence of the topic categories “venom characterisation” and “biological activity”, the two most studied topic categories overall (93.91% and 59.90% of all analysed publications, respectively; Figure 4).

Despite the very relevant and topical issue of snakebite and the antivenom crisis [50,51,52], the topic categories “antivenomics and neutralisation” and “envenomation symptoms” are underrepresented in our analyses (Figure 4). However, considering that the aim of this study was to analyse the general trends in snake venom research, we believe this potential underrepresentation to be due to our keywords not directly addressing antivenom studies and envenomation reports. Nevertheless, we were able to detect an increase in the curves relative to these categories starting from the first half of the 2010s (see Figure 6). This is concordant with several publications and awareness campaigns which, together with the very recent official recognition of snakebite as a neglected tropical disease by the World Health Organization [53], have recently been addressing the human health burden of snakebite and the antivenom crisis [3,10,12,37,54], renewing the interest in snake venom research in general and likely stimulating the study of these topics.

The topic category “prey specificity of venom” was, after “envenomation symptoms”, the least studied one (Figure 4), but the curve relative to it underwent a significant increase over the past few years. While acknowledging the possible presence of topic biases determined by our article search criteria, we believe that the very low number of articles covering this category might be due to the current neglect of this topic. Indeed, toxinological research has been slow to embrace the importance of focusing on the relationship between snake venom and prey to improve the understanding of the drivers behind snake venom evolution and variation (e.g., diet-related venom variation), and help the development of therapies against snakebite [26,27]. In light of this, we suspect the recent rise we detected in the number of articles considering the topic category “prey specificity of venom” to be most likely linked to the general increase in snake venom studies rather than to the beginning of a change in trend.

The curves relative to the categories “interspecific venom variation”, “geographic venom variation”, and “individual venom variation”, all falling within the field of comparative venomics, follow very similar trends, presenting a considerable increase over the last two decades (Figure 6). Interestingly, the combination of the topic categories “interspecific venom variation” and “venom characterisation” is the combination most frequently encountered across the analysed articles (Figure S1), indicating that a consistent amount of them likely focused on the comparison of the venoms of different snake species. Taken together, these results suggest that most of the analysed studies likely opted for the application of a comparative approach, and that this might have become even more relevant in recent years.

## 4. Conclusions

We acknowledge the possible presence of methodological limitations in this work. Specifically, the exclusive use of Google Scholar for article search, in combination with the article selection criteria applied, likely excluded some potentially relevant publications from our analysis. Nevertheless, our results are in line with trends and biases in snake venom studies already reported in the previous literature [15,28,38], and are thus to be considered reliable. We detected an overall positive trend, with a consistent increase since the early 2000s in the number of snake venom studies published yearly, even more evident over the last decade. Nevertheless, our analysis also highlighted a consistent neglect of snake families of supposedly minor medical relevance (e.g., Homalopsidae, Psammophiidae, Pseudoxyrhophiidae), an apparently limited focus on some of the areas most impacted by snakebite (i.e., Asia and Africa), and potentially minor interest in the ecological and functional context of snake venom. The study and characterisation of the venoms produced by other venomous snake taxa, excluding the typically more studied families (i.e., Colubridae, Elapidae, Viperidae), should be implemented in future snake venom studies in order to increase the knowledge about snake venom evolution and composition, and help widen the spectrum of treatable snakebite envenomations. Additionally, more effort should be put into developing studies focusing on species originating from the areas where snakebite incidence is high and the economical level low, such as the Indomalayan and the Afrotropic realms, which appear to be underinvestigated. Finally, snake venom should be analysed taking into account ecological and functional contexts of the species producing it, in order to pave the way to obtaining a more detailed and comprehensive view of the driving forces behind snake venom evolution and variation. We hope that by providing a qualitative and quantitative estimate of the taxonomic, geographic, and topic biases present in snake venom research, our work will be useful to define a road map for future efforts aiming at focusing on the most glaring knowledge gaps in this field of study.

## 5. Materials and Methods

### 5.1. Article Selection

Publications considered for the current study were gathered and organised using the Google Scholar (Google Inc. (Menlo Park, CA, USA)) web search engine (https://scholar.google.com), between the months of December 2018 and March 2022. To perform the search, the following query was used, applying every possible combination of the ten selected keywords: (<venom> OR <venomics>) AND (<toxin> OR <composition> OR <profile>) AND (<snake> OR <viper> OR <elapid> OR <colubrid> OR <atractaspid>). The evaluation timeframe we defined went from 1964 to 2021. Search results were sorted by relevance following Google Scholar default search options, with the quality of the result-search match being higher on top of the result list and progressively decreasing. We thus reviewed for consideration the first 200 articles obtained for each keyword combination searched, checking their suitability for inclusion in the final dataset. Articles focusing on the study of snake venom composition and variation, presenting either a protein-centred venom approach or an indirect approach based on different techniques (e.g., transcriptomics, bioinformatics, toxicity assays) were taken into account for analysis. Articles not investigating whole snake venoms (e.g., reviews, publications focusing only on single venom fraction analysis, single toxin studies), and/or not published in refereed, impacted journals were not considered.

The following information was recorded from each article: (i) publication year, (ii) taxonomy of the analysed species, (iii) country and biogeographic realm of origin of the analysed specimens, and (iv) topics covered.

### 5.2. Taxonomic Information

In order to assess what the most studied and most represented snake taxa were, information about family, subfamily, genus, and species of the specimens analysed in each article was collected. Due to phylogenetic uncertainty within the family Elapidae [55,56,57], we did not consider subfamilies for this group, but instead divided it into two main categories widely used in the literature, irrespective of concerns over monophyly [58,59,60]: (i) Old World and American elapids and (ii) Australo-Papuan and marine elapids. The retrieved taxonomic information was updated mainly following the taxonomy reported by The Reptile Database [2], based on information about species names and sampling localities of the specimens. When insufficient locality and taxonomic information did not allow the unambiguous identification of the analysed snake species, we kept the specific IDs as reported in the original articles.

### 5.3. Hazard Categories

In order to test whether the harmful potential of a species’ venom could influence eventual biases in terms on number of studies dedicated to it, we developed a hazard index based on the existing bibliography (e.g., [11,40]), WHO guidelines (e.g., [4,33,34]), and authors’ opinion. We classified the snake species considered in the retrieved studies into four categories, based on the severity of the envenomation they can cause: (i) category 1—“critical clinical relevance”: envenomations have a generally high chance to cause death or significant disability if professional medical care is not obtained; (ii) category 2—“high clinical relevance”: envenomations usually cause significant illness, hospitalisation is required, death and/or disability are unlikely but possible if professional medical care is not obtained; (iii) category 3—“moderate clinical relevance”: envenomations are unpleasant but typically not life-threatening, significant disability is exceptional, typically treated symptomatically; (iv) category 4—“low clinical relevance”: envenomations likely cause only very mild symptoms (e.g., local swelling, itching, limited blistering), generally not interfering with normal activities and not being life-threatening, and professional medical care rarely necessary. Species we could not assign to any of the abovementioned categories were classified as “unknown” and not included in the analyses.

### 5.4. Origin of the Specimens

Information about the country where each snake species that produced the analysed venom samples came from, and the corresponding biogeographic realm, was also gathered and used to assess possible geographical biases in snake venom studies. Country and biogeographic realm of origin of specimens for which information about the place of origin was ambiguous or unavailable (e.g., captive specimens, pooled venoms) were considered as “unknown” and not included in the analyses. Biogeographic realms were identified following the RESOLVE Ecoregions 2017 website [61].

### 5.5. Topics Covered

In order to identify the most investigated research topics in the retrieved articles, we gathered information about the research topics covered in the reviewed publications, and grouped them into eight categories: (i) “venom characterisation”: defining the composition of the venom of snake species through the application of one or more techniques, from basic venom fractionation to “omic” approaches (i.e., proteomics, transcriptomics, genomics); (ii) “antivenomics and neutralisation”: evaluating immunological mechanisms in model animals and/or efficacy of one or more antivenoms against the venom of the analysed snake species; (iii) “biological activity”: assessing the enzymatic, toxic, and/or lethal (i.e., LD50) activity of the venom produced by the analysed snake species; (iv) “envenomation symptoms”: description of envenomation symptoms in humans resulting from snakebite accident; (v) “geographic venom variation”: comparing venom profiles, components, and/or biological activity between individuals belonging to the same snake species but coming from different populations and/or habitats across their natural range; (vi) “individual venom variation”: comparing venom profiles, components, and/or biological activity between individuals of the same snake species, with a focus on venom variation related to differences in age (i.e., ontogeny), sex, and/or diet; (vii) “interspecific venom variation”: comparing profiles, components, and/or activity of venoms produced by snakes belonging to different species; (viii) “prey specificity”: testing efficacy and/or efficiency of the venom of the analysed snake species against the preferred natural prey.

### 5.6. Chronological Trends

Information about the publication year of each analysed article was gathered in order to define the total number of publications per year, and thus identify the most and least productive years in terms of published articles. Using this information, we built cumulative curves in order to identify trends in terms of studied families, subfamilies, and research topics varied across the retrieved articles along the considered timeframe. The data obtained this way allowed to assess patterns of chronological variation in the above-mentioned categories.

### 5.7. Statistical Analyses

We performed chi-squared (*χ*^2^) tests to assess the significance of the differences in terms of article coverage detected between snake taxa (i.e., family, subfamily, genus), countries, biogeographic realms, and topic categories. To investigate the presence of significant relationships between number of publications on snake venom and years from 1964 to 2021, we tested the following regression models: (i) 1st order polynomial, (ii) 2nd order polynomial, and (iii) 3rd order polynomial. We ranked the models on the basis of the corrected Akaike’s Information Criterion (AICc) [62], ultimately applying the model with the lowest AICc score considered as the best-fitting one. We applied the same method to also choose the best model to test the presence of significant relationships between the number of years that passed from 1964 to 2021 and the number of yearly papers covering each of the eight topic categories defined.

To investigate whether family, hazard category, and biogeographic realm of origin of the snake species retrieved from the analysed articles could be correlated with the number of articles dedicated to each one of them, we used Generalised Linear Models (GLM) assuming a Poisson distribution for the response variable. Country of origin and subfamily were excluded from the used predictors because the retrieved information relative to them was often fragmentary and ambiguous, and because they were nested in the predictors “biogeographic realm” and “family”, respectively. Collinearity between the three predictors considered (i.e., family, hazard category, biogeographic realm) was low (Variance Inflation Factors (VIF) always <5.11), thus we included all of them in the regression models generated. We built the models using the number of articles dedicated to each species as response variable, and all possible combinations of the three predictors considered. The produced models were ranked on the basis of their AICc score, considering the model with the lowest AICc score as the best-fitting one.

Polynomial regression models were generated using the software SPSS (version 13.0. [63]). All other analyses were performed in R environment (version 4.1.1 [64]). We used the packages *vegan* [65] and *MuMIn* to build the full set of Generalised Linear Models [66].

## Figures and Tables

**Table 1:** Number of snake species assigned to each of the four hazard categories defined by family and subfamily/group. The percentages refer to the total number of species retrieved for the considered taxonomic category/group from the publications analysed.

		Hazard Category				
	N Species	Cat. 1	Cat. 2	Cat. 3	Cat. 4	Unknown
**Viperidae**	144	73 (50.69%)	65 (45.14%)	6 (4.17%)	0	0
Azemiopinae	1	0	1 (100%)	0	0	0
Crotalinae	109	55 (50.46%)	54 (49.54%)	0	0	0
Viperinae	34	18 (52.94%)	10 (29.41%)	6 (17.65)	0	0
**Elapidae**	110	55 (50%)	33 (30%)	15 (13.64%)	6 (5.45%)	1 (0.91%)
Australo-Papuan and marine elapids	58	25 (43.1%)	12 (20.69%)	14 (24.14%)	6 (10.35%)	1 (1.72%)
Old World and American elapids	52	30 (57.69%)	21 (40.39%)	1 (1.92%)	0	0
**Colubridae**	35	2 (5.71%)	2 (5.71%)	12 (34.39%)	19 (54.39%)	0
Ahaetuliinae	1	0	0	0	1 (100%)	0
Colubrinae	28	2 (9.1%)	0	8 (36.4%)	12 (54.5)	0
Dipsadinae	4	0	0	4 (50.0%)	4 (50.0%)	0
Natricinae	2	0	2 (50.0%)	0	2 (50.0%)	0
**Atractaspididae**	4	0	4 (100%)	0	0	0
**Homalopsidae**	2	0	0	0	2 (100%)	0
**Psammophiidae**	2	0	0	1 (50.0%)	1 (50.0%)	0
**Pseudoxyrhophiidae**	1	0	0	0	1 (100%)	0
**Total**	298	130 (43.62%)	104 (34.9%)	34 (11.41%)	29 (9.73%)	1 (0.34%)

**Table 2:** Final set of Generalised Linear Models (GLM) tested. The models relate the number of articles dedicated to each snake species retrieved in the analysed articles with the selected independent variables: family, biogeographic realm, and hazard category. The best-fitting model is reported in bold. The table reports the number of parameters in the model (K), the information score of the model (Akaike’s Information Criterion corrected for small sample sizes; AICc), the difference in AICc score between the best model and the model being compared (ΔAICc), and the AICc weight (i.e., the proportion of the total amount of predictive power provided by the full set of models contained in the model being assessed; *w*AICc).

Model	K	AICc	ΔAICc	*w*AICc
**Biogeographic realm and Hazard category**	10	1058.710	0.000	0.991
Hazard category	4	1068.161	9.450	0.009
Biogeographic realm	7	1110.483	51.772	5.675 × 10^−12^
Family	8	1115.132	56.421	5.552 × 10^−13^
*Null*	1	1116.503	57.792	2.797 × 10^−13^

## Data Availability

The data presented in this study are available in this article and supplementary material.

## Supplementary Materials

The following supporting information can be downloaded at: https://www.mdpi.com/article/10.3390/toxins14120884/s1, Table S1: Complete dataset of the articles analysed in the present work; Table S2: Complete list of all retrieved snake species; Table S3: Country of origin of the retrieved snake species; Table S4: Number of publications considered per each year of the defined time frame; Table S5: Polynomial regression models applied to test the relationship between topic categories and number of yearly papers covering each one of them; Table S6: Summary of the Generalised Linear Model (GLM) applied to test the relationship between number of articles retrieved for each snake species and hazard category (HAZARD) and biogeographic realm (REALM); Figure S1: Information about the combinations of topic categories retrieved in the analysed articles; Figure S2: Chronological trends of the analysed publications, by snake subfamily/group considered.
