# Supplementary material for: Toxic Habits: An Analysis of General Trends and Biases in Snake Venom Research
Source: Toxins (Basel). 2022 Dec 17;14(12):884. doi: 10.3390/toxins14120884 (PMC9783912; doi:10.3390/toxins14120884)
Supplement: Supplementary file 1 [file toxins-14-00884-s001.zip › toxins-2042675-supplementary ms/Supplementary Figures.pdf]

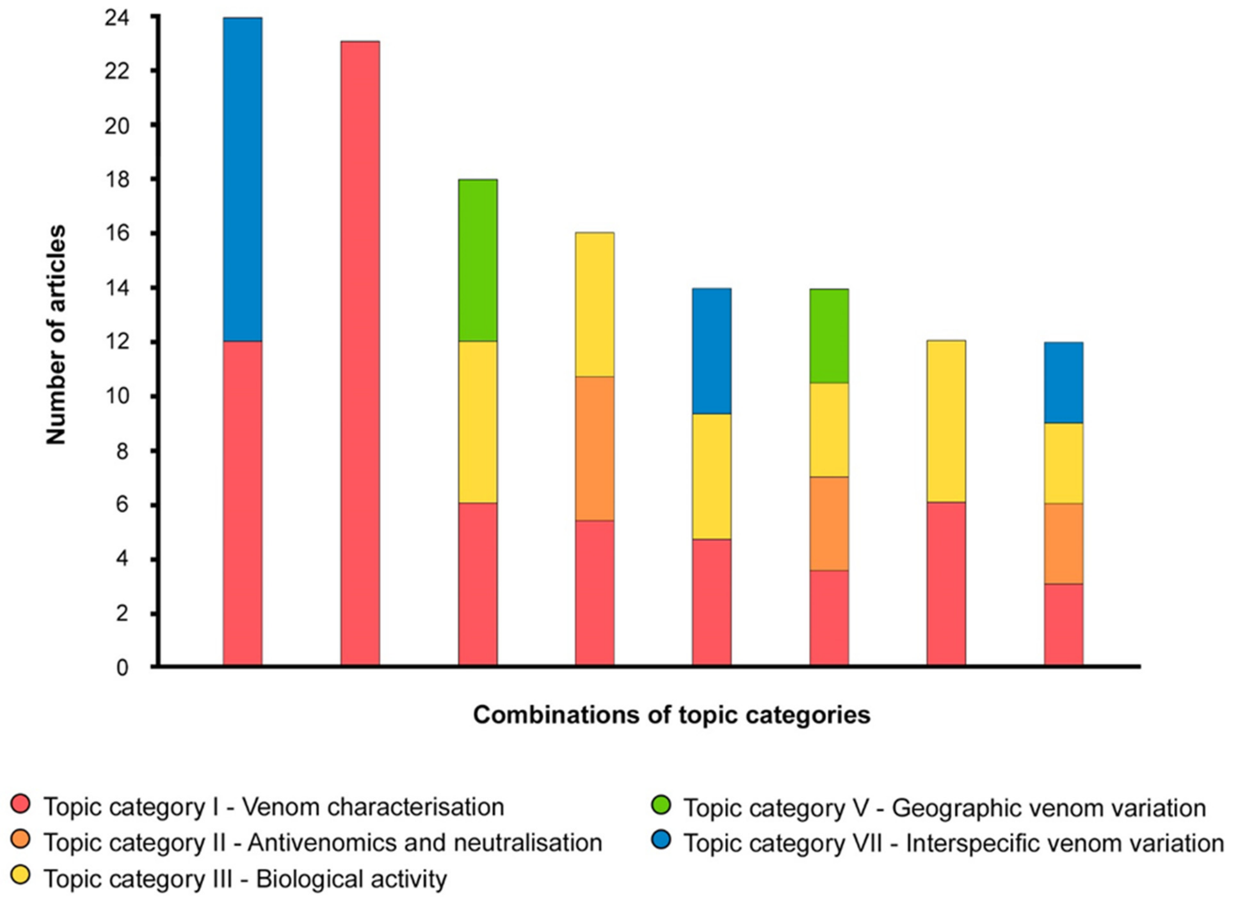

Figure S1. Information about the combinations of topic categories retrieved in the analysed articles.

The graph shows the eight most represented combinations in terms of number of articles.

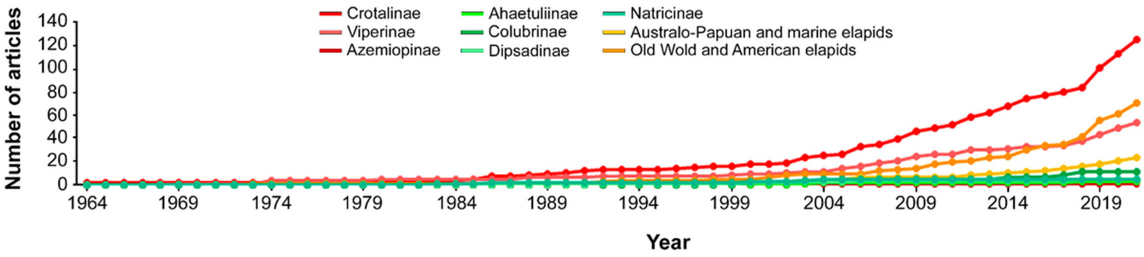

Figure S2. Chronological trends of the analysed publications, by snake subfamily/group considered. Notice the prevalence of Crotalinae, Viperinae, and Old World and American elapids over the other subfamilies/groups.
